# Association between Prenatal and Postnatal Psychological Distress and Toddler Cognitive Development: A Systematic Review

**DOI:** 10.1371/journal.pone.0126929

**Published:** 2015-05-21

**Authors:** Dawn Kingston, Sheila McDonald, Marie-Paule Austin, Suzanne Tough

**Affiliations:** 1 Faculty of Nursing, University of Alberta, Edmonton, Alberta, Canada; 2 Faculty of Medicine, University of Alberta, Edmonton, Alberta, Canada; 3 Faculty of Medicine, University of Calgary, Calgary, Alberta, Canada; 4 St. John of God Health Care, University of New South Wales, Burwood, Australia; 5 Alberta Centre for Child, Family and Community Research, Calgary, Alberta, Canada; University of Rennes-1, FRANCE

## Abstract

**Purpose:**

Maternal psychological distress is one of the most common perinatal complications, affecting up to 25% of pregnant and postpartum women. Research exploring the association between prenatal and postnatal distress and toddler cognitive development has not been systematically compiled. The objective of this systematic review was to determine the association between prenatal and postnatal psychological distress and toddler cognitive development.

**Methods:**

Articles were included if: a) they were observational studies published in English; b) the exposure was prenatal or postnatal psychological distress; c) cognitive development was assessed from 13 to 36 months; d) the sample was recruited in developed countries; and e) exposed and unexposed women were included. A university-based librarian conducted a search of electronic databases (Embase, CINAHL, Eric, PsycInfo, Medline) (January, 1990-March, 2014). We searched gray literature, reference lists, and relevant journals. Two reviewers independently evaluated titles/abstracts for inclusion, and quality using the Scottish Intercollegiate Guideline Network appraisal tool for observational studies. One reviewer extracted data using a standardized form.

**Results:**

Thirteen of 2448 studies were included. There is evidence of an association between prenatal and postnatal distress and cognitive development. While variable effect sizes were reported for postnatal associations, most studies reported medium effect sizes for the association between prenatal psychological distress and cognitive development. Too few studies were available to determine the influence of the timing of prenatal exposure on cognitive outcomes.

**Conclusion:**

Findings support the need for early identification and treatment of perinatal mental health problems as a potential strategy for optimizing toddler cognitive development.

## Introduction

Maternal mental health problems are among the most common morbidities in pregnancy and postpartum with up to 25% of women experiencing stress, depression, or anxiety [1,2]. The increased risk of early neonatal outcomes (e.g., preterm birth and maternal-infant attachment) associated with poor prenatal and postnatal maternal mental health is well-established [3]. However, 50 to 70% of women with anxiety and depression in pregnancy develop chronic symptoms that continue while they are parenting children in their early years, and thus may have an enduring impact on child development [4–6]. Notably, a recent study (N = 1,507) identified that children born to the 30.5% of women in the sample with sub-clinical depression symptoms from pregnancy to 4 years scoring 6–8 on the Edinburgh Post Natal Depression Scale (EPDS), as well as those born to the 8.8% of women with persistently high and increasing depression symptoms across this period (EPDS scores 10–14) had significantly higher rates of emotional-behavioural difficulties (19.1% and 23.9%, respectively) compared to children of women with minimal symptoms (7.0%) [7].

Historically, the main focus in perinatal mental health has been the adverse effects of postpartum depression. However, epidemiologic evidence of rates of prenatal anxiety and depression that are similar or greater than postnatal rates [8], new developments in neuroscience, and the epigenetic consequences of early, adverse experiences [9], have stimulated interest in the influence of maternal mental health across the continuum of the perinatal period, the capacity of the postnatal environment to ameliorate risk incurred prenatally [10,11], and their ultimate impact on long-term child outcomes, particularly child development [12].

Inquiry regarding specific mechanisms linking maternal psychological distress to suboptimal child outcomes is in the early stages [13,14]. Longitudinal evidence from prospective studies supports fetal programming (changes to the fetal neurologic system under the influence of an adverse intrauterine environment that create vulnerability for poor lifelong health and development) as a likely mechanism underlying the association between prenatal psychological distress and sub-optimal child development [8,14,15]. Based on several studies from the Avon Longitudinal Study of Parents and Children (ALSPAC), an attributable load of 10–15% of prenatal anxiety and depression on emotional-behavioural problems has been estimated [14,15]. Programming may result from increased fetal exposure to cortisol through impaired placental function that reduces metabolism of cortisol and/or increases serotonin, or increased inflammatory cytokines related to increased stress [14]. Evidence also supports a strong epigenetic influence involving the interaction between prenatal biological influences and the postnatal environment [10,11]. Indeed, a recent study reported the first human evidence linking prenatal stress with long-term epigenetic changes in the form of DNA methylation in 8-year old children [16].

Toddlerhood is considered to be a critical developmental period [17]. It is a time of increased sensitivity to epigenetic mechanisms because the brain is undergoing rapid neuronal and synaptic proliferation, myelination, and hippocampal development, contributing to differential responses to stress as a consequence of early adversity in children under 3 years of age compared to older children [9,18]. Healthy cognitive development during toddlerhood is of primary interest across health, education, and social sectors because it influences future academic performance and mental health well into adulthood, as revealed by large birth cohort studies [19,20]. Furthermore, cognitive development issues frequently co-occur with problems in other developmental domains (e.g., socio-emotional, behavioural) and together can have a ‘cascading’ effect, negatively impacting other areas of development during toddlerhood and extending into adolescence [21].

Cognitive development is strongly dependent upon early experiences [19,22]. While some studies have demonstrated that maternal psychological distress adversely influences toddler cognitive development, others have not. General reviews suggest that the association exists [15,23]; however, a rigorous systematic review of this evidence has not been conducted. A recent review of eleven studies of prenatal stress and anxiety and child cognitive outcome reported a small negative association in children from birth to 5 years of age [24]. Given the sensitive period that toddlerhood represents, the lifelong importance of optimal toddler development, the lack of an existing review on prenatal depression on cognitive development, and the need to compare prenatal and postnatal maternal mental health on cognitive development, there is a need for, a comprehensive study of the association between prenatal and postnatal mental health and toddler cognitive development to guide practice and policy related to early detection, prevention, and intervention.

The objective of this systematic review was to summarize evidence on the relationship between prenatal and postnatal maternal psychological distress and cognitive development in toddlers. It extends the work of a previous systematic review that we conducted on the association between maternal psychological distress and infant development (birth to 12 months) [13]. Because we wanted to determine the impact of prenatal and postnatal psychological distress across time by stage of child development, the present study builds on the previous review by examining the association between maternal distress and one aspect of toddler development—cognitive development. In this review, we hypothesized that a statistically significant association exists between both prenatal and postnatal psychological distress and cognitive development in toddlers, whereby children of mothers who experienced high levels of prenatal or postnatal psychological distress would have poorer cognitive outcomes than those children whose mothers experienced no or low levels of distress.

## Methods

### Inclusion and Exclusion Criteria

Observational studies of any type (case-control, cohort, cross-sectional) were included in this review if they evaluated the association between the most common forms of maternal psychological distress and toddler cognitive development as characterized by: (a) stress, depression, or anxiety occurring during pregnancy or the postpartum period (1 year following birth); (b) an outcome measure of cognitive development from 13 months of age up to and including 36 months; (c) recruitment of participants from developed countries; (d) publication in English between January, 1990 and March, 2014; and (e) inclusion of women exposed and unexposed to prenatal and/or postnatal psychological distress. Studies were excluded if the initial measure of maternal distress was beyond 1 year postpartum, psychological distress was based on a physiologic measure (e.g., cortisol), or the sole exposure was a pharmacologic treatment for maternal distress.

### Definitions

Maternal psychological distress was defined as stress, depression, or anxiety occurring during the prenatal or postnatal period. We classified maternal distress as prenatal if it occurred during pregnancy, and postnatal if it occurred from delivery to one-year post-delivery.

The trimesters of pregnancy were categorized as first (0–13 weeks gestation), second (>13 weeks–26 weeks), and third (>26 weeks–40+ weeks). The magnitude of effect was defined as small when the correlation (r) was 0.10–0.30 or the odds ratio (OR) was <1.7; as medium when r = 0.30–0.50 or OR>1.7 to 2.5; and as large when r ≥ 0.50 or OR>2.5 [25].

### Search Strategy and Study Selection

A university-based librarian developed the search strategy. Five electronic databases (Embase, CINAHL, Eric, PsycInfo, Medline) were searched. The main MeSH keywords were: pregnancy; postpartum period; depression; anxiety; psychological stress; mental health; child development; and cognitive. Reference lists of included articles and relevant reviews were examined and key journals were hand-searched. We also searched government sites and private agencies with information on child development for unpublished and gray literature (e.g., Public Health Agency of Canada, Alberta Family Wellness Initiative). The search encompassed the period from January, 1990 to March, 2014. Two individuals independently reviewed each article’s title or abstract with resolution of disagreements through discussion and consensus. Reviewers coded each article as included or excluded based on inclusion criteria, and further coded excluded articles by reason for exclusion.

### Quality Assessment

Two independent reviewers appraised the quality of each article using the Scottish Intercollegiate Guideline Network (SIGN) tool for observational studies (http://methodology/checklists.html). The SIGN tool is widely used and recommended for quality appraisal of cohort, cross-sectional and case-control studies (http://www.sign.ac.uk/index.html). Each study was appraised based on six components: study design, potential for selection bias, confounders, withdrawals and dropouts, blinding (e.g., of outcome assessor), and measurement of exposures and outcomes. Based on an algorithm designed for the Quality Assessment Tool for Quantitative Studies (Effective Public Health Practice Project, http://www.ephpp.ca/tools.html), each component was defined as strong, moderate, or weak with an overall study quality assigned based on the total number of strong, moderate, or weak components. Studies were categorized as having an overall quality of strong if no components were designated as *weak* and at least four components were *strong*, moderate if one component was *weak* and less than four components were *strong*, and weak if a rating of *weak* was assigned to two or more components. Disagreements regarding quality ratings were resolved by consensus.

### Data Extraction

Study data were abstracted by one reviewer using a standardized data extraction form designed for this review, and verified for accuracy by a second reviewer. Decisions regarding the selection of data to abstract were based on factors that could influence the association between maternal distress and cognitive development (e.g., demographics, outcome assessor, type and timing of distress) and quality of the evidence (e.g., measures, potential confounders). Authors were contacted regarding missing data. Completed data extraction forms are available from the authors.

### Analysis and Ethics

We planned a qualitative (descriptive) analysis of the findings and examined studies for the potential to conduct a meta-analysis. If heterogeneity across studies was low, we planned on conducting a fixed-effect meta-analysis. In the presence of heterogeneity, we intended on using a random-effects meta-analysis. This review was exempt from ethics review board approval.

## Results

### Overview of Studies Table 1

**Table 1:** Key Aspects of Studies Included in Review (n = 14).

Citation/ Quality	Study Design and Sample	Measure and Timing of Primary Exposure	Infant outcome (assessor)	^a^Results (effect size)	Adjusted for key potential confounders
**STUDIES WITH PRENATAL EXPOSURE**					
Bergman et al., 2007[27] (N = 123) Quality: moderate	Longitudinal cohort (UK)-amniocentesis clinic; mean age 36.55 years; 82.9% Caucasian	Prenatal stress (SLEQ) (assessed retrospectively at 16–19 months postpartum)	Cognitive development at 17 months (BSID, MDI) (researcher)	S (Number prenatal stressful life events β = -.47, p<.01) (medium) NS (Number postnatal stressful life events)	S: education; child age NS: maternal age; prenatal smoking and alcohol; child sex; current depression/ state-trait anxiety; social support; stress -17 months
Brouwers et al., 2001[29] (N = 131) Quality: strong	Longitudinal cohort (Netherlands)-community-mean age 30.4; mean years education 10.8	Prenatal anxiety (STAI) (32 weeks; prospective)	Cognitive development at 2 years (BSID, MDI) (researcher)	S (prenatal anxiety predicted MDI score; B = -.33, p = .003) (medium)	S: education; child sex NS: Apgar scores; HOME score; age; parity; smoking/alcohol; delivery; breastfeeding; birth weight; gestational age; PPD at 12 months; current depression
DiPietro et al., 2006[31] (N = 82) Quality: moderate	Longitudinal cohort (United States)-community-mean age 31.8 years; mean years education 17.0	Prenatal stress, anxiety, depression (STAI; PES; POMS; DSI; PSS; CES-D) (24–32 weeks; prospective)	Cognitive development at 2 years (BSID, MDI) (psychologist)	Prenatal Exposure: S (composite of anxiety (β = 2.59, p<.05) & depression (β = 2.46, p<.05) (medium) NS (stress) Postnatal Exposure: NS (depression, anxiety, stress)	NS: postnatal (6 weeks) & current anxiety, depression, stress; education; infant sex; birth weight; gestational age
Laplante et al., 2004[34] (N = 52) Quality: moderate	Longitudinal cohort (Canada)-community-8.6% ≤high school; 12.1% unskilled occupation; 12.0% <$40,000 income	Prenatal stress (Objective stress; Subjective stress- IES-R; retrospective) (all trimesters)	Intellectual abilities at 2 years (BSID, MDI) (researcher)	Objective stress: S (1^st^ trimester exposure: β_unstd_ = -.52, p<.05; β_std_ = -.52, p<.05) (small) S (2nd trimester: β_unstd_ = -.74, p<.01; β_std_ = -.64, p<.05) (medium) NS (3^rd^ trimester) Subjective stress—NS	NS:Gestational age S:Birth weight (trait anxiety; PPD, obstetric complications not significant in correlational analysis and not included in regression) (S:timing of exposure moderator)
Laplante et al., 2004[34] (N = 52) Quality: moderate	Longitudinal cohort (Canada)-community-8.6% ≤high school; 12.1% unskilled occupation; 12.0% <$40,000 income	Prenatal stress (Objective stress; Subjective stress- IES-R; retrospective) (all trimesters)	Intellectual abilities at 2 years (BSID, MDI) (researcher)	Objective stress: S (1^st^ trimester exposure: β_unstd_ = -.52, p<.05; β_std_ = -.52, p<.05) (small) S (2nd trimester: β_unstd_ = -.74, p<.01; β_std_ = -.64, p<.05) (medium) NS (3^rd^ trimester) Subjective stress-NS	NS:Gestational age S:Birth weight (trait anxiety; PPD, obstetric complications not significant in correlational analysis and not included in regression) (S:timing of exposure moderator)
Tse et al., 2010[35] (N = 990) Quality: strong	Longitudinal cohort (United States)-community-mean age 32.6 years; 74% college; 90% annual income ≥$40,000	Prenatal depression (EPDS) (mid-pregnancy; Mean weeks 27.9; SD 2.0; prospective)	Child cognition at 3 years (Peabody Picture Vocabulary; Wide Range Achievement of Visual Motor Abilities) (researcher)	NS (at EPDS cut-off of 13 or above and 15 or above)	Education; race; age; parity; income; pregnancy intention; partnership status/education; Alcohol/smoking; anxiety; gestational age; birth weight; child sex; support; breastfeeding; employment; daycare; PPD (significance not reported)
Zhu et al., 2014[32] (N = 152) Quality: strong	Longitudinal cohort (China)-community-mean age 27 years	Prenatal stress (PLEC) (retrospectively reported at 32–34 weeks for first trimester) (PLEC)	Cognitive development (BSID, MDI) at 16–18 months (psychologist)	S (0–14 weeks gestation) (adjusted M/SD 103.11/11.06 vs 110.09/9.80, F_(5,146)_ = 14.50, p<.001) (large)	Postnatal depression; prenatal smoking and alcohol maternal and paternal; gestational age; birth weight; breastfeeding (significance not reported)
**STUDIES WITH POSTNATAL EXPOSURE**					
Aiello et al., 2007[26] (N = 71) Quality: weak	Longitudinal cohort (Australia)-mean age 18.3 years; 67.6% < high school; 66.2% Caucasian	Postpartum maternal separation anxiety (MSAS) (6 & 12 months postpartum; prospective)	Cognitive development at 15 months (BSID, MDI) (psychologist)	NS	S: Separation-individuation NS: perceptions of being parented; attachment; maternal IQ; age; pregnancy history; living with parents; verbal intelligence
Cornish et al., 2005[28] (N = 112) Quality: moderate	Longitudinal cohort (Australia)-parentcraft centre; mean age 31.4 years; 11% ≤high school; 93% Caucasian	PPD (CES-D; CIDI) (4 & 12 months; prospective)	Mental development at 15 months (BSID, MDI) (researcher)	NS (briefly depressed vs never depressed) S (chronically depressed vs never depressed, OR 3.36, p<.025) (large) (chronic vs brief not assessed)	S:Gender NS: Education; maternal age; mother bilingual
Murray et al., 1992[38] (N = 111) Quality: moderate	Longitudinal cohort (UK)-community-mean age 28.0 years; 40% manual labour or unemployed; all partnered	Postpartum depression (EPDS, SPI) (6 weeks, 6 & 12 months; prospective)	Cognitive development at 18 months (Object concept task; BSID, MDI) (researchers)	Object concept task: S (in depressed mothers with and without previous history compared to never depressed, X^2^ = 4.22, df = 1, p<.04 [small]; no difference between mothers with PPD +/- history) MDI: NS	NS: Gender; education; unplanned pregnancy; obstetric complications; marital friction; quality of social relationships; housing; employment; paternal psychiatric history; parity; birth status; birth weight; current stressful life events S: social class
Piteo et al., 2012[36] (N = 360) Quality: moderate	RCT (control group only) (Australia)—community 30–38% ≤high school	Postnatal depression (6 weeks, 6 months; prospective)	Cognitive development at 18 months (BSID, MDI) (psychologist)	NS	NS: Gestational age; occupation; education; history depression; social support; breastfeeding S: home environment
Sutter-Dallay et al., 2011[37] Quality: moderate	Longitudinal cohort (France)—community mean age 29.6 (SD 4.2), 72% education ≥12 years	Postnatal depression (EPDS) (6 weeks, and 3, 6, 12, 18, 24 months; prospective)	Child cognitive development (BSID, MDI) (18 and 24 months) (psychologists)	S (6 weeks) with MDI at 18 month and 24 month (p = .007); NS-depressive symptoms over 2 year period (p = .19)	NS: Child sex; age; education; income; parity
**STUDIES WITH BOTH PRENATAL AND POSTNATAL EXPOSURES**					
Koutra et al.,2013 [30] (N = 223) Quality: moderate	Longitudinal cohort (Greece)-community-12.6% low education	Prenatal anxiety (STAI) (28–32 weeks); Prenatal and postnatal depression (EPDS) (prenatal 28–32 weeks; postnatal 8 weeks; prospective)	Cognitive development at 18 months (BSID, MDI) (psychologists)	Prenatal anxiety (NS) Prenatal depression (S) (β_std_ = -5.45, 95% CI -10.44, -0.46) (adjusted for postnatal depression) Postnatal depression (S) (β_std_ = -5.80, 95% CI -11.65, -0.05) (adjusted for prenatal anxiety/ depression)	Child sex; quality of assessment; gestational age; maternal age; education; breastfeeding duration (significance not reported)

^a^Results reported as adjusted when available. Effect sizes reported when available or calculable

Note. NS = non-significant (p≥.05); S = significant (p<.05); PPD = postpartum depression; CES-D = Center for Epidemiological Studies; CIDI = The Composite International Diagnostic Interview DSI = Daily Stress Inventory; EPDS = Edinburgh Postnatal Depression Scale; GHQ = General Health Questionnaire; IES-R = Impact of Event Scale-Revised; MSAS = Maternal Separation Anxiety Scale; PES = Pregnancy Experiences Scale; POMS = Profile of Moods Scale; PSS = Perceived Stress Scale; SLEQ = Stressful Life Events Questionnaire; SLE: Stressful life events; PLEC = Prenatal Life Events Checklist; SPI = Standardized Psychiatric Interview; STAI = State-Trait Anxiety Inventory; BSID, MDI = Bayley Scales of Infant/Toddler Development, Mental Development Index

**Fig 1:**
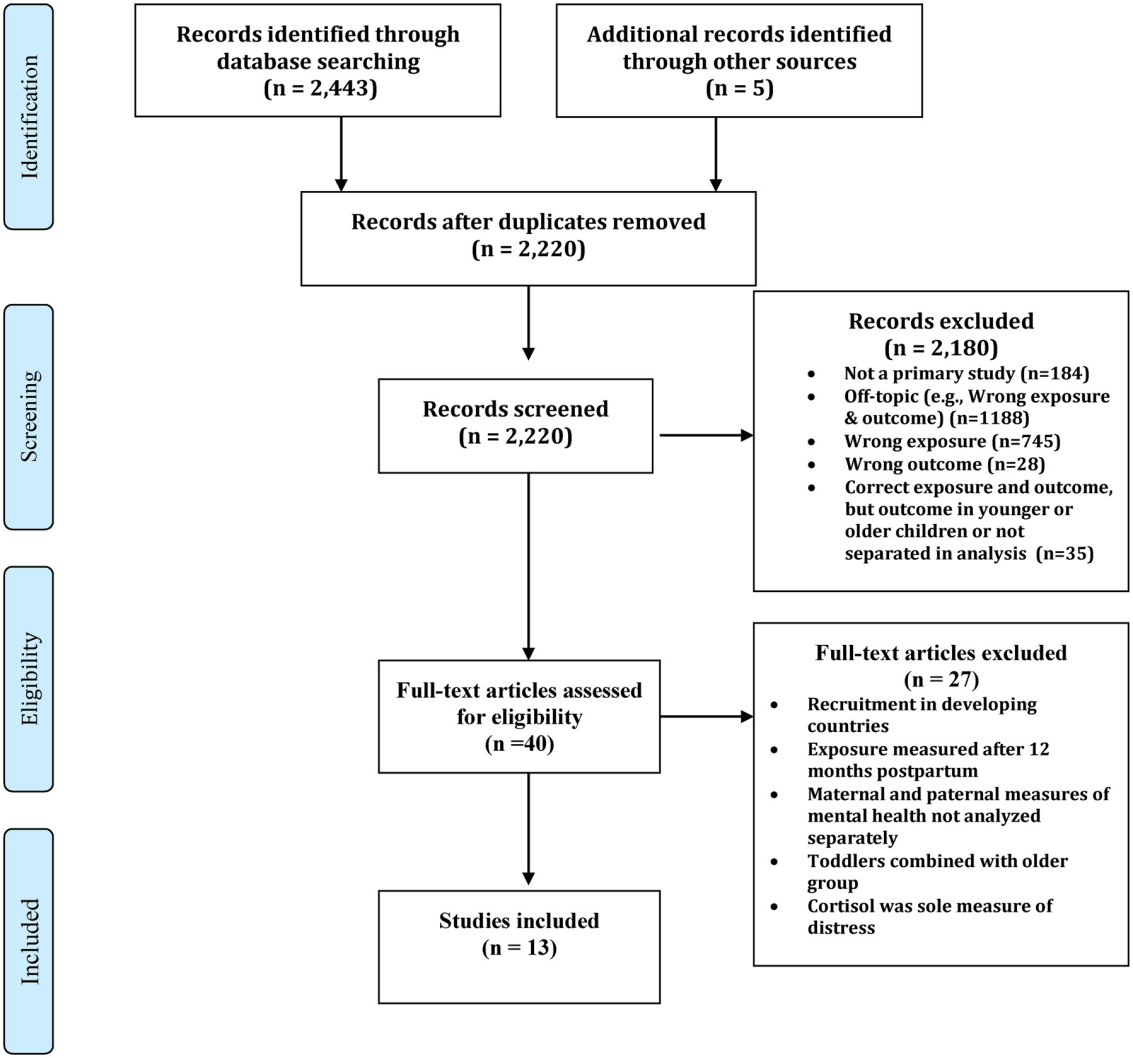
Flow Diagram.

A total of 2,448 studies were captured by the search with 13 studies meeting inclusion criteria (Fig 1). The list of excluded articles is available from the authors. Eight countries were represented, including the United States (US) (n = 2), United Kingdom (UK) (n = 2), Canada (n = 2), Netherlands (n = 1), Australia (n = 3), France (n = 1), China (n = 1), and Greece (1). Thirty-eight percent of the studies (n = 5) were published during the past 5 years. All studies used longitudinal designs. Ten studies recruited community-based samples. Of the remaining three studies, one recruited adolescents [26], one included women attending an amniocentesis clinic [27] and one recruited women residing at a residential centre for infant difficulties [28]. We were unable to conduct a meta-analysis because of the wide diversity of toddler outcome measures (e.g., dichotomous and continuous measures) and missing data that could not be retrieved from study authors related to maternal exposure status.

Eight studies examined prenatal distress as the primary exposure [27,29–35], and six studied postnatal distress [26,28,30,36–38]. (Note. Koutra et al. examined *both* prenatal and postnatal distress as primary exposures). In terms of timing of exposure, one measured prenatal distress during the first trimester [32], one during the second trimester [35], two during the third trimester [29,30], one during both the second and third trimesters [31], and three across all trimesters [27,33,34].

Fig 1

### Summary of Quality of Studies

Among the thirteen studies, three had overall quality ratings of strong [29,32,35], nine were rated as moderate [27,28,30,31,33,34,36–38], and one was weak [26] (Tables 1 and 2). Lower quality ratings were primarily due to attrition and not assessing potential confounders that, by definition, may be related to both maternal distress and cognitive development (e.g., prenatal smoking and alcohol use, breastfeeding) (Table 2).

**Table 2:** Summary of Quality of Studies Included in Review (N = 13).

Citation		Components of quality appraisal								Controlled for exposure to distress at other times (✓ X = no; PE = primary exposure)		
	Prenatal or Postnatal Exposure	Design	Selection Bias	Confounders	Withdrawals	Blinding	Measurement	^a^Overall study quality	^b^Effect Size	Prenatal	Postnatal	Current
Bergman et al., 2007[27]	Prenatal	S	M	S	W	S	S	M	Med	PE	X	✓
Brouwers et al., 2001[29]	Prenatal	S	M	S	S	S	S	S	Med	PE	✓	✓
DiPietro et al., 2006[31]	Prenatal	S	M	W	M	S	S	M	Med	PO	✓	✓
Laplante et al., 2004[34]	Prenatal	S	M	M	S	W	S	M	Sm/ Med	PE	✓	X
Laplante et al., 2007[33]	Prenatal	S	M	M	S	W	S	M	Med	PE	✓	X
Tse et al., 2010[35]	Prenatal	S	M	S	M	S	S	S	NS	PE	✓	X
Zhu et al., 2014[32]	Prenatal	S	M	S	S	S	S	S	L	PE	✓	X
Aiello et al., 2007[26]	Postnatal	S	M	W	M	W	S	W	NS	X	PE	X
Cornish et al., 2005[28]	Postnatal	S	M	W	S	S	S	M	L	X	PE	X
Murray et al., 1992[38]	Postnatal	S	M	W	S	S	S	M	Sm	X	PE	✓
Piteo et al., 2012[36]	Postnatal	S	W	M	S	S	S	M	NS	X	PE	X
Sutter-Dallay et al., 2011[37]	Postnatal	S	M	M	W	S	S	M	NC	✓	PE	✓
Koutra et al., 2013[30]	Both	S	M	S	S	W	S	M	NC	PE	PE	X

Note. Only studies reporting statistically significant associations included in table.

^a^For quality: S = strong, M = moderate, W = weak

^b^For effect sizes: Sm = small, Med = medium, L = large, NS = non-significant; NC = not calculable;

Given that prenatal psychological distress is one of the strongest predictors of postnatal psychological distress, it should be considered when evaluating the influence of postnatal distress on child outcomes [39,40]. Current distress is important to measure for two key reasons, including: 1) the need to account for its association with cognitive development so that the independent effects of prenatal and postnatal mental health can be parsed out; and 2) when mothers are reporting on their child’s development, its negative influence on her perception of development (i.e., reporter bias) [41]. However, a concern across the field of perinatal epidemiology remains the nature of how potentially intermediary factors [42], such as postnatal and current distress, are addressed. When considering the association between prenatal distress and child outcomes, it is plausible that postnatal and current distress are on the pathway between exposure and outcome such that the influence on the child outcome may be through these intermediary influences. Thus, simply controlling for these potential intermediary influences may introduce a paradoxical result or a bias that underestimates the magnitude and direction of relationship[42] between prenatal distress and child outcomes. Among the studies with prenatal exposure, two controlled for both postnatal and current distress [29,31], five controlled for postnatal distress alone [30,32–35], and one controlled for current distress alone [27] (Table 2). Among the six studies with a primary postnatal exposure, one controlled for current distress [38], one controlled for prenatal distress [30], and one controlled for both [37] (Table 2). Our analysis revealed that a main strength of the studies in this review was the use of researchers or psychologists as assessors of the child outcome, thus lessening the potential for reporter bias that could lend to a spurious association.

A wide variety of instruments were used to measure maternal psychological distress in these studies, and all studies scored ‘strong’ in the subcategory of ‘measurement’ for using tools with demonstrated reliability and validity (Table 2). All instruments utilized maternal self-report to provide exposure data, with one study supporting the self-report tool with a diagnostic interview [28]. All studies used psychometrically evaluated measures of toddler cognitive development, with twelve of thirteen studies using Bayley Scales of Infant Development.

### Main Findings

Overall, ten of thirteen studies reported significant associations between maternal distress and cognitive development.

#### Prenatal Distress

Seven of the eight studies evaluating prenatal distress as a primary outcome reported significant associations—six with negative associations and one with a positive association [31]. Among these seven significant studies, all were of moderate quality [27,29–31,33,34], with the exception of Zhu et al. (2014) [32], which scored as strong. The largest study with a primary prenatal exposure (n = 990, quality strong) did not show a significant association with prenatal depression after controlling for multiple potential confounders, including postnatal depression symptoms [35]. In terms of prevalence, one study reported rates of cognitive delay that were roughly three times greater in toddlers born to women with prenatal anxiety distressed women (22% [29]) compared to non-distressed women (6% [29]).

Among the studies with reported or calculable effect sizes, five of seven significant studies of prenatal distress and toddler cognitive development found medium effect sizes [27,29,31,33,34]. Zhu et al. (2014) reported large effect sizes. (Note: Laplante et al., 2004 found a small effect for first trimester and medium for second trimester [34]).

#### Postnatal distress

Four of the six studies of postnatal distress [28,30,37,38] had significant findings with all scoring as moderate in quality. The single study that reported rates of cognitive delay found that were over three times greater in toddlers born to women with postnatal depression (29% [28]) compared to those without (9%[28]). Among the three significant postnatal studies with reported or calculable effect sizes, effect sizes included small [38], medium [31], and large effects [28].

#### Types and Timing of Maternal Exposure

Specific types of distress and their timing of measurement are summarized in Table 3. Four studies assessed prenatal distress retrospectively [29,32–34]. Each assessed objective stress retrospectively and was significant with moderate [29,33,34] and strong [32] effect sizes. Significant associations were found between cognitive development and prenatal anxiety (2/3 studies), prenatal objective stress (4/4), and prenatal depression (2/3). There is less evidence for an association with subjective stress based on one significant study where subjective stress related to a past disaster was assessed [33]. Postnatally, most studies (4/5) reported a significant association between postnatal depression and cognitive outcomes. However, conclusions regarding the impact of postnatal anxiety are unclear based on one non-significant study of separation anxiety conducted in adolescents [26].

**Table 3:** Summary of Association with Cognitive Development by Timing and Types of Psychological Distress.

	Association with Cognitive Development and Effect Size	
Prenatal	Significant (Effect size^b^)	Non-significant
Anxiety	Brouwers et al., 2001[29] (M); DiPietro et al., 2006[31] (M)	Koutra et al., 2013[30]
Subjective stress	Laplante et al., 2007[33]^**a**^ (M)	DiPietro et al., 2006;[31] Koutra et al., 2013;[30] Laplante et al., 2004[34]^**a**^
Objective stress	Bergman et al., 2007[27]^**a**^ **(M)**; Laplante et al., 2004[34]^**a**^ (S/M); Laplante et al., 2007[33]^**a**^ (M); Zhu et al., 2014[32]^**a**^ (L)	No studies
Depression	DiPietro et al., 2006[31] (M); Koutra et al., 2013[30] (NC)	Tse et al., 2010[35]
**Postnatal**		
Anxiety	No studies	Aiello & Lancaster, 2007[26]
Depression	Cornish et al. 2005[28] (L); Koutra et al., 2013[30] (NC); Murray, 1992[38] (S); Sutter-Dallay et al., 2011[37] (NC);	Piteo et al., 2012[36]

^**a**^ **retrospective measurement of distress**

^**b**^ **effect size noted for significant studies only L = large; M = medium; S = small; NC = non-calculable**

In terms of timing of exposure, significant relationships were found with cognitive development when maternal distress occurred in the first [34], second [34] and third trimesters [29], across the second and third trimesters [31], during ‘pregnancy’[27,33], and during the postnatal period [28,38]. The only study that systematically evaluated the impact of timing across all three trimesters in the same cohort found a significant association between objective stress (due to a natural disaster) and cognitive development when pregnant women were exposed during the first and second trimesters, but not the third [34]. Thus, there were too few studies to draw definitive conclusions about whether timing of exposure influences cognitive outcomes.

## Discussion

Among the 2,448 studies captured through the search, 13 studies were eligible for inclusion in this review. The small number of studies evaluating the association between maternal psychological distress and development in toddlers reflects the relatively understudied nature of the preschool period compared to infancy and school-age [43], and our conclusions must be interpreted in this context. However, overall our conclusions stem from well-conducted, prospective longitudinal studies of moderate-strong quality across eight countries. Seven of eight studies of moderate-strong quality suggest that there is an association between prenatal psychological distress and cognitive development in toddlers, with all demonstrating a negative association except one; similarly, four of six studies of moderate quality reported significant negative postnatal associations.

The findings of this review are consistent with a growing body of evidence that suggests that exposure to prenatal distress can negatively influence children’s development through complex biological pathways including neuroendocrine changes [12,15,44–47], and epigenetic mechanisms [10]. The positive association between prenatal anxiety and depression and MDI scores reported by DiPietro et al. (2006) raises important questions. The investigators posit that the relationship between distress and cognitive outcomes may be U-shaped, rather than linear. In other words, mild distress (as in this study) may optimize neuronal development [31]. Another main consideration is whether unmeasured protective factors moderated the effect, since only infant sex and maternal education were included in the analyses as potential confounders.

We did not observe a pattern in the relationship between specific forms or timing of prenatal distress and cognitive development. The lack of a clear pattern may suggest that there is little specificity regarding the association between unique types of distress and cognitive development. However, the associations across heterogeneous types of distress may also reflect comorbidity of mental health problems (e.g., stress, anxiety, depression) that is common in pregnant and postpartum women [48], but not considered in these studies.

Of importance, two studies examined the influence of chronicity of depression symptoms on cognitive development. Cornish et al. (2005), found that proportions of infants with sub-optimal MDI scores were similar in women with brief postpartum depression (symptoms at 4 months, not at 12–15 months) and no depression (10%), whereas infants of mothers with chronic depression (postpartum depression at 4, 12–15 months) were over 3 times more likely to have poor cognitive development [28]. Conversely, Sutter-Dallay et al. (2013) did not find an association between postpartum depression measured at 3, 6, 12, 18, and 24 months and MDI scores (p = .19) [37]. One possible explanation may that Cornish et al. recruited women from a residential centre for help with infant problems, whereas Sutter-Dallay et al. recruited women from low-risk maternity clinics. The diverse approaches to the measurement of chronic depression may also contribute to different results.

One of the unanticipated findings of this review was how few additional predictors of toddler cognitive development were significant once maternal mental health was considered in statistical models, although studies assessed a wide variety of demographic, socioeconomic, obstetrical, maternal behavior, neonatal and child factors. Within this context, the association between toddler development and maternal distress found in this review underscores its importance as an early life risk factor. Future research regarding the relationship between prenatal and postnatal distress and child development should address two key questions: 1) What protective prenatal influences and interventions lessen the impact of prenatal psychological distress on poor child outcomes; and 2) What postnatal influences and interventions minimize or reverse the adverse neurological sequelae of a sub-optimal intrauterine environment (e.g., due to maternal psychological distress)? Notably, the research of the past two decades represented in this review largely explores intrauterine risk factors without also examining potential protective factors that occur during the prenatal or postnatal periods.

### Strengths and Limitations

This systematic review summarized evidence from observational studies published during the past 24 years on the association between maternal psychological distress and toddler cognitive development. However, the limitations of this review and the body of literature on which it was based should be acknowledged. The conclusions of the review are based on a small number of studies (n = 13). We did not include articles published in languages other than English or prior to 1990. The prevalent missing data related to maternal exposure (e.g., proportion of children with or without cognitive delay by maternal exposure), the inability to retrieve missing data, and the diversity in reporting of outcomes by continuous and dichotomous outcomes precluded meta-analysis. To address these limitations, future studies should report proportions of children with and without the outcome by maternal exposure. Several studies did not control for potential confounders that influence toddlers (e.g., language spoken at home, educational programs attended). Some potential for misclassification bias exists as a result of measuring maternal psychological distress at one time or with a single self-report instrument [49]. Thus, women who experience situational distress may be classified as ‘distressed’, but are essentially ‘false positives’—women who are identified by the instrument as having distress when in fact they do not. Thus, the study may find an association with cognitive development when one does not exist (Type I error) or the magnitude of the true association is overestimated. Most articles did not report pertinent details of maternal distress, such as severity or treatment. Therefore, we could not explore their impact on toddler development.

### Implications and Conclusions

While based on a limited number of studies (n = 13), the conclusions of this review from primarily community-based studies point inevitably to consideration of early prenatal and postnatal detection and intervention of perinatal mental health problems on a population-level. Maternal mental health is a modifiable risk factor. Emerging evidence suggests that early intervention aimed at reducing maternal mental health problems in the prenatal and postnatal periods is effective at improving maternal mental health, maternal functioning, parenting, child cognitive development, and maternal-child interaction [50–52]. The need for maternal mental health screening as a component of routine well-child visits has been acknowledged by the American Academy of Pediatrics [53] and the Canadian Paediatric Society [54]. Similarly, routine prenatal mental health screening has been endorsed by the Association of the College of Obstetricians and Gynecologists [55] and other major national [53,56,57] and international [17,58] organizations. However, maternal mental health problems remain severely underdetected and undermanaged in both pediatric [8,59] and obstetrical care [60]. Without standardized screening and treatment, over 50% of pregnant women with depression and anxiety [6] will experience chronic symptoms through their child’s early years [4]. Given that women see an obstetrical care provider on average fourteen times during pregnancy and postpartum [61], and a paediatrician or family physician seven times through well-child visits [62], one of the earliest and potentially most impactful approaches to risk reduction of child cognitive delay may be to embed effective, feasible, and sustainable approaches to maternal screening, referral, and treatment as components of routine obstetrical and well-child care [8,63]. Finally, the small number of studies found in this review reveal that further research is needed to disentangle prenatal and postnatal risk and protective factors on toddler cognitive development, the mechanisms that underlie the association, and the mitigating influence of the postnatal environment on prenatal risk.
